# Mobile-Based Ecological Momentary Interventions for Grief in China and Switzerland: Protocol for a Collaborative and Iterative Qualitative App Development Study

**DOI:** 10.2196/87021

**Published:** 2026-06-09

**Authors:** Ayala Sara Licht-Handler, Chenchen Lin, Eva-Maria Stelzer, Clare Killikelly

**Affiliations:** 1 Department of Psychology, University of Zurich, Binzmühlenstrasse 14, Zürich, 8050, Switzerland, 41 0446357441

**Keywords:** ecological momentary interventions, grief, bereavement, mobile phone, culture, death

## Abstract

**Background:**

Bereavement is a common and inevitable part of life. However, it is also difficult and disruptive. Prolonged grief disorder has recently been added to the *International Classification of Diseases, 11th Revision*, and the *Diagnostic and Statistical Manual of Mental Disorders (Fifth Edition)*. Grief is a deeply cultural experience; however, most research about grief and grief intervention is conducted in Western, educated, industrialized, rich, and democratic (WEIRD) countries. Support for grief is often limited and difficult to access. We propose that ecological momentary interventions (EMIs) present an opportunity to widen access to grief support and develop culturally relevant interventions, given EMIs’ easy accessibility and opportunity to offer self-help support in people’s natural environments.

**Objective:**

This study aims to describe the development protocol of 2 EMIs for grief, one in China and one in Switzerland. he EMIs are intended for individuals seeking additional grief support without a diagnosis of prolonged grief disorder. The EMIs will be developed to be culturally relevant and appropriate for each country; therefore, contextual factors may prescribe slightly different protocols to fit the needs of each cultural group.

**Methods:**

Both interventions will be developed using a collaborative research approach. This means that the content for the app will be developed after consultation with grief experts, bereaved participants (potential end users), and the research team. After the initial content development, another round of feedback will be gathered to ensure acceptability.

**Results:**

Funding for both studies has been secured through a grant in 2024. The scoping phase for both WEIRD and non-WEIRD contexts has been completed. At the time of submission, both studies have started recruitment, and the Chinese study has conducted interviews with 17 bereaved participants. The next steps are to continue recruitment and data gathering, analyze the collected data, and extract important themes for intervention components, and then begin the app content development. Results are expected by the end of 2026 and will subsequently be prepared for publication.

**Conclusions:**

The study presents 2 similar but nonidentical development protocols for EMIs for grief support in 2 countries, where one is a WEIRD country and the other is a non-WEIRD country. Similarities and differences in the developmental process across both countries are discussed, along with challenges associated with adapting grief interventions into an EMI format.

## Introduction

### Background

Grief and bereavement are common and inevitable parts of life [1,2]. While most people adjust to life after bereavement without needing professional support [3], the experience of grief can be very difficult and disruptive [4]. Typical grief reactions include sadness, longing, and feelings of emptiness and nostalgia [5].

Mourning is a complex process, with grief often being described as arriving in waves or pangs [6,7]. In addition to these shifts of grief over time, grief has been shown to fluctuate throughout people’s everyday lives [8]. This is in line with the dual process model of coping with bereavement [9], which suggests that bereavement is characterized by oscillation between loss-oriented and restoration-oriented work. This oscillation occurs in the absence of a predefined pattern and in a nonlinear order.

Grief has come into increased focus following the COVID-19 pandemic and the inclusion of prolonged grief disorder (PGD) into the *International Classification of Diseases, 11th Revision* [10], in 2018 and the *Diagnostic and Statistical Manual of Mental Disorders (Fifth Edition)* [11] in 2022 [4]. Approximately 5% to 10% of bereaved adults in Western, educated, industrialized, rich, and democratic (WEIRD) countries were reported to develop PGD following the death of a loved one, with slightly higher rates reported in non-WEIRD countries, particularly in China [12,13].

PGD is characterized by intense grief reactions, such as emotional numbness, avoidance of reminders of the loss, yearnings for the deceased, and feeling that life since the loss is meaningless, persisting for a prolonged period after the bereavement and disrupting daily functioning [3,10].

### Grief and Culture

Grief is a universal experience; however, mourning is inherently shaped by culture [14]. Culture influences the way people express their grief, show help-seeking behaviors, receive social support, understand the meaning of mourning symptoms, and engage with coping rituals [15,16]. Nevertheless, most research on grief, PGD, and grief therapy stems from WEIRD countries, with most research originating from the United States [4].

Grief symptoms are expected to vary between individualistic and collectivist societies [17]. One variation across cultures is the expected duration of mourning. For example, in Germany, a mourning period of 1 year (the *Trauerjahr*) is the cultural norm. In contrast, some Chinese traditions expect mourning periods of up to 3 years for the loss of a child or parent, with some memorial activities persisting for one’s lifetime [14,18,19]. This variation is recognized by the *International Classification of Diseases, 11th Revision*, which specifies that acute grief reactions must persist for “an atypically long period of time following the loss, markedly exceeding expected social, cultural or religious norms for the individual’s culture and context” to diagnose PGD.

A study looking at Chinese and Swiss bereaved parents concluded that the features of PGD were substantiated in both samples; however, some differences emerged [17]. Swiss parents showed more preoccupation with the loss, whereas Chinese parents reported more meaninglessness, depressive symptoms, and higher functional impairment. In addition, higher comorbidity rates between PGD and depression or anxiety were found in Chinese samples than Western ones [19]. Chinese individuals might additionally report more somatic symptoms (eg, headaches, stomach aches, or back pain) [20,21] and might culturally prefer to suppress rather than express grief-related emotions [12].

### Current Challenges With Grief Support

A tiered model of bereavement care has been suggested to address care needs across different levels [22]. This model describes 4 tiers. Tier 0 includes public education, tier 1 consists of universal and community-based support, tier 2 refers to targeted nonspecialist interventions, and tier 3 includes specialist mental health support.

While specialist grief support is available, it is often reserved for those with clinical levels of PGD (tier 3), leaving many people struggling with bereavement (tiers 1 and 2) without support [23,24], resulting in a large treatment gap [25]. Lack of knowledge, perceived stigma, and inappropriate support further reduce access rates to already insufficient services [24,26]. Minority groups report additional barriers in accessing grief support [27]. In addition, few mental health professionals have been trained in grief therapy or grief counseling, leading to a lack of provision [26]. This issue is particularly pronounced in China, where a severe shortage of mental health professionals has been reported alongside concerns regarding stigma and limited mental health literacy [28].

In WEIRD countries, there seems to be a shift away from religious and traditional ceremonies and more toward new ways of mourning (eg, alternative rituals, online commemoration, and virtual tombs) [29,30]. In Switzerland, this transition has influenced grieving practices by moving away from primarily religion-based mourning traditions. This shift signals the need for new ways to support bereaved individuals.

### Digital Grief Interventions

Mental health mobile apps promise convenience, accessibility, anonymity, reduced stigma, and flexibility [31]. Online bereavement support has therefore been suggested to close access gaps [23]. Indeed, online-based interventions exist across all pyramid tiers [32], and significant positive effects of online grief interventions have been reported [33,34]. Multiple recent reviews have found that online interventions for grief seem feasible, acceptable, and effective in supporting bereaved people [33,35,36], with one systematic literature review and meta-analysis even suggesting lasting effects after 3 months [36]. The authors suggest that this might be particularly due to the inclusion of cognitive behavioral therapy (CBT) techniques. This is aligned with reviews citing that CBT is the most common basis for grief online interventions [35,37].

Compared to web-based interventions, mobile-based interventions offer the additional benefits of providing real-time monitoring and feedback as well as easier accessibility [31]. A new class of interventions called ecological momentary interventions (EMIs) could be particularly well suited for individuals who have lost a loved one and have subclinical grief levels. EMIs provide support in people’s everyday life (in real time) and natural settings (in the real world) [38]. The rationale is similar to therapists encouraging people to practice skills in between appointments and apply them in their lives [38]. EMIs typically work by providing short “microinterventions” during the day (eg, a message or a small task) [39]. This has the benefit that information and skills get practiced and integrated into real life and that support can be accessed anywhere [40].

EMIs have increasingly gained popularity, particularly following the widespread adoption of smartphones [40,41]. A systematic review of EMIs has found that EMIs for common mental health disorders have been successfully used in both healthy and clinical populations [42]. EMIs could therefore be a particularly useful tool to offer app-based support for people experiencing nonclinical grief. However, a systematic search of apps on the app store revealed that only 6.2% of apps for depression and anxiety, which claimed that they use a theoretical evidence-based framework, actually published evidence for the apps’ efficacy [43], thus emphasizing the importance of well-tested interventions.

There are already some existing grief support apps and EMIs. Help Texts (formerly known as Grief Coach) is a texting-based EMI for grief support at tier 1 [44]. Help Texts can send text-based notifications to a griever and supporters who have signed up. The messages are based on CBT and acceptance and commitment therapy, and include different strategies such as psychoeducation, acknowledgment and validation, emotional and instrumental support, coping skills, and grief work. Help Texts nowadays includes a variety of protocols for different types of grief [45]. Grief Coach has been tested in the United States and United Kingdom, where it was perceived as helpful and demonstrated high retention rates [44,46]. However, both studies did not include measures of intervention effectiveness.

*My Grief* is a Swedish app-based intervention for bereaved parents with elevated symptoms of prolonged grief [47] and an example of tier 2 support (eg, [32]). The Swedish CBT-based intervention offers psychoeducation, self-monitoring, CBT-based exercises, and signposting [48]. The authors reported reductions in both grief symptoms and posttraumatic stress symptoms following the intervention, although the observed effect sizes were small [48].

More than ever, there is a need for low-cost, accessible, and evidence-based grief support. EMIs offer an alternative to traditional therapeutic models and may be easily adaptable to different cultures and contexts.

### Our Study

Our study seeks to build on these findings by developing EMIs for grief in Switzerland and China, targeting the general population of individuals experiencing grief who are seeking additional support (tiers 1 and 2), rather than focusing on a specific subpopulation. This approach reflects our goal of creating apps that, if proven effective, could be integrated into real-world care and made accessible to potential end users. Currently, most apps developed in research settings end up not being used commercially [49], and even among commercially available apps, user retention rates tend to be low [50]. This means that current practices do not offer sustainable interventions with potential for long-term use. We hope that by developing an app for tiers 1 and 2 of the pyramid (information about bereavement and nonspecialist support), we can offer support to a wide pool of potential end users. We have therefore decided not to focus on a specific subpopulation, similar to much of the grief literature (such as self-help books), which is typically aimed at grieving individuals in general.

The apps are based around a CBT framework of grief support [51] and the dual process model of coping with bereavement [9]. CBT for grief has been chosen as it has a strong evidence base [52], has been previously successfully used for online interventions for grief [35,37], and the structure of CBT lends itself well to the EMI format. In addition, the CBT-based nature, focused on adapting behaviors, is assumed to help participants to learn to externalize some of their internalized grief reactions (eg, through writing or sharing of the experience). Externalization is a key mechanism of change in psychotherapeutic theory and may be a core process that leads to integrated and adaptive grief [53].

The rationale behind EMI posits that continued prompted practicing of skills in a person’s real life will enable them to better learn to use the skills [38]. Similar to classical CBT skills, which encourage people to practice target exercises daily, EMIs provide prompts for in-the-moment use, which can encourage participants to practice skills at different times in an accessible online format. EMI prompts are also expected to increase motivation compared to module-based online interventions. EMI could be particularly well suited for grief, as avoidance has been identified as a core topic in maladaptive grief. EMI prompts can challenge this avoidance by suggesting different exercises that gently challenge avoidance throughout the day. In line with the dual processing model, we expect the apps to include both restoration and loss-oriented exercises. While the loss-oriented exercises can challenge emotional avoidance, the restoration-oriented exercises can challenge themes of experiential avoidance. Through the prompts, participants are encouraged to try different exercises (loss oriented and restoration oriented) at different times of the day and in different contexts, exploring their effects on their mood. In addition, the prompts encourage repetition and practice of exercises. The underlying theory of change for experience sampling interventions suggests that by helping individuals track their thoughts, feelings, and behaviors in real time, this may increase self-awareness of patterns and triggers.

Additionally, self-monitoring (through ecological momentary assessment [EMA]) contributes to insight about grief-related emotions [54,55] and counters the tendency to overgeneralize feelings in grief [56]. EMA can additionally support the understanding of which contextual factors influence current emotions and, in turn, facilitate adaptive coping. As grief can vary greatly throughout the day [8], this could help foster insight about patterns and understanding of the change in emotions.

The other mechanisms of change in the app include psychoeducation, which is expected to help normalize and destigmatize different grief reactions and foster acceptance [57,58].

Nevertheless, while this is our theoretical background for the apps, we remain open to inputs from experts and bereaved individuals in guiding the content of the intervention. Accordingly, different themes and exercises may be considered relevant and helpful across different cultural contexts.

### Objectives

The objectives of the presented studies are to develop 2 interventions to support people on tiers 1 and 2 in the bereavement care pyramid. This is likely to include individuals experiencing grief without the diagnosis of PGD, who nevertheless feel like they could use additional nonspecialist support or information relating to grief [22]. This target group has been chosen as it covers a wide section of the bereaved population [59], and there remains a lack of accessible support for those without a diagnosis of PGD and access to specialist psychological support [23,24]. In addition, exposure is a core component of CBT for grief [51,60]. However, this is more difficult to implement by using an EMI format, as microinterventions are typically very short and therefore not suited for full-length exposure. Thus, while people with PGD might enjoy the app or find it useful, they might benefit more from targeted specialist support.

One study will develop the intervention in Switzerland and one in China. This will include multiple components: identifying the appropriate intervention content, turning the content into microinterventions for just-in-time use (or optional background), and designing a delivery plan for the intervention components. In addition, we will use EMA items alongside the intervention to investigate user well-being while using the apps.

## Methods

Both app interventions will be developed using m-Path [61], a platform for EMA and EMI studies [62]. m-Path is accessible for phones through either Google Play Store (Android phones) or the App Store (iOS phones).

### Data Protection

The m-Path app does not collect personal data. For use of the app, users enter a pseudonym. The app collects no demographic or personal data. However, the app can collect potentially sensitive information (eg, EMA data). Data from m-Path is hosted on Microsoft Azure servers in Germany, which are compliant with General Data Protection Regulation international standards such as International Organization for Standardization 27001 and System and Organization Controls 2. m-Path’s entire data security policy can be found in Multimedia Appendix 1.

### Ethical Considerations

Ethics approval for the development of studies has been sought from Beijing Normal University Institutional Review Board (Nr: BNU202501090008) for the Chinese study and the University of Zürich Ethics Commission (Nr: 24.12.10) for the Swiss study.

All bereaved adults will receive and sign informed consent forms before the interviews or focus groups. Participants are informed that their data will be recorded, transcribed, and then deidentified. Participants will also be informed that only deidentified data will be published.

All bereaved adults will be offered compensation for their participation. Participants in the Swiss focus groups will be reimbursed 30 CHF (1 CHF=US $1.29) for participating in the focus groups. In addition, participants can get reimbursement for any travel costs for in-person meetings. In the Chinese study, each participant receives 100 CNY (1 CNY=US $0.15) after completing the interview.

### Collaborative Design

Both studies will develop the interventions in collaboration with people who have experienced bereavement. The development process will follow user-centered design and participatory design [63]. User-centered design focuses on combining the expertise of researchers with the lived experience of potential end users through a collaborative process of cocreating interventions.

**Figure 1.**
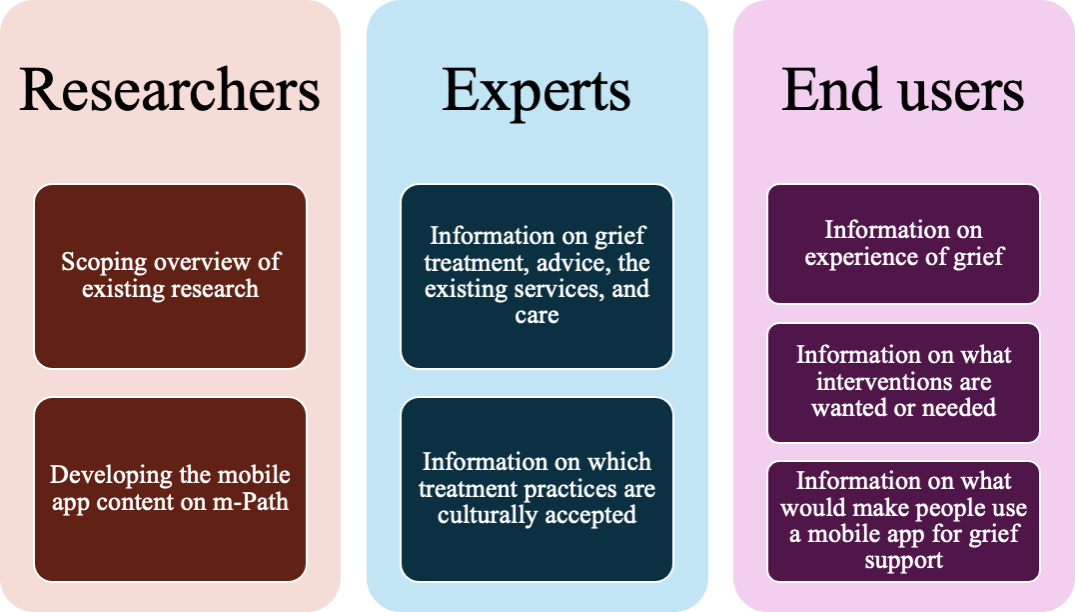
Cocreation responsibilities.

In the presented interventions, 3 groups will be included in the cocreation: potential end users, grief experts, and researchers. In our case, the researchers will also be the ones physically developing and creating the app using m-Path. The distinct cocreation responsibilities are illustrated in Figure 1. Both interventions will also be developed within their cultural contexts, involving researchers, end users, and experts from the target settings.

The app development process will include three stages: (1) scoping, (2) end-user consultation and app development, and (3) beta testing [64]. We address the first 2 parts here.

### Iterative Process

Both studies will use iterative processes in their app development. Iterative process refers to cycles of developing, testing, and refining an app [65]. This can be particularly useful when cocreating with multiple groups with different levels of expertise, as it allows adjustments to be made for unexpected challenges, overlooked elements, or outcomes that differ from initial expectations. In addition, a postdevelopment feedback round allows for feedback on aesthetic and practical aspects that might impact app use.

### Scoping

Presently, as far as we are aware, there are no established guidelines or protocols for grief EMIs. Therefore, as a first step, we will conduct systematic reviews in WEIRD and non-WEIRD contexts to document existing internet-based interventions and app-based grief interventions.

In WEIRD contexts, extensive systematic reviews and meta-analyses on bereavement care and internet-based interventions already exist (eg, [32,66]). Therefore, in the Swiss context, we will conduct a systematic literature review of intervention components of just-in-time adaptive interventions for common mental health disorders.

In the Chinese context, a systematic review and meta-analysis of grief interventions in non-WEIRD countries will be conducted. This topic was chosen as most grief research is based in WEIRD countries [4] and therefore cannot simply be extrapolated to the Chinese context.

### Expert Interviews

Various experts will be consulted on recommended intervention components for the apps. We aim to draw on a broad range of expertise to reflect different knowledge about grief support in China and Switzerland.

The expert interviews will include questions regarding recommendations of specific psychoeducation and intervention components to be included in the app, as well as how to best adapt such interventions into the microintervention format for EMI. EMI exercises typically consist of short text-based components or audio exercises that last a few minutes [40]. Interviews will also ask about key components identified in the literature (eg, need for community and social connection). Both interview guides for Swiss and Chinese contexts can be found in Multimedia Appendix 2.

In the Swiss study, experts invited to one-on-one interviews include experts on CBT for grief (research and practice), psychotherapists, people working in palliative care, chaplains, and people from the grief support charity sector. These experts were chosen to represent various types of expertise: (1) research expertise in grief interventions—researchers, (2) treatment expertise in grief intervention—psychotherapists and clinical psychologists, and (3) experts in grief support through long-standing grief support experience—people leading self-help groups, palliative care members, and chaplains. All Swiss experts are also treated as experts in the local context and asked specifically about “Swiss” experiences and needs for adaptation.

In the Chinese context, experts in grief, clinical psychology, and EMA are invited. All invited experts are Chinese and will be treated as experts in grief treatment within this context. In addition, a more formalized iterative cultural adaptation will be conducted with the expert focus group. As no EMA items exist in Chinese for this population, the EMA items used in the app will also be developed with the Chinese experts.

Both the Swiss and the Chinese studies are aiming to recruit up to 10 experts for the interviews (Switzerland) or focus group (China). Experts are recruited through the research teams’ professional network as well as by emailing relevant experts found online.

In line with the iterative process of app development, we will request feedback from the experts once the app content has been developed.

### End-User Consultations

End-user consultations are used to assess the experience of grief and grief support, as well as the needs and preferences of the target population. Key cultural experiences and expressions of grief are explored, as well as attitudes and experiences with online platforms. Part of the interviews are based on the Bereavement and Grief Cultural Formulation Interview [67]. Participants were also asked to reflect on key components identified through research (such as CBT) or expert interviews, such as culturally relevant rituals and self-coping strategies, and issues related to platform development.

In the Chinese study, end-user consultation takes the format of one-on-one online interviews with bereaved adults. The aim is to recruit 20 participants. For this, purposive sampling is carried out. Participants who experienced bereavement will be recruited. The sampling accounts for grief scores [68], gender, age, type of loss, and duration of bereavement. The purpose of this is to obtain a more representative sample of general bereaved individuals.

For Switzerland, a focus group with bereaved adult participants will be conducted. We aim to recruit bereaved Swiss adults who have been bereaved, consider themselves affected by grief, are fluent in German (speaking, reading, and writing), live in Switzerland, and would use a smartphone for grief support. No questionnaires regarding grief severity will be used to reflect potential real-world use. We aim to recruit an equal number of men and women across a range of age groups to better understand potential differences in needs and preferences. End users will be recruited through flyers and online advertisements, as well as through self-help groups, social support groups, community centers, and psychological practices. Participants will be screened and excluded if they are in a current mental health crisis and require acute psychological support. We aim to recruit approximately 20 participants, which is what we consider feasible with our current resources.

The end users who participated in the app development will be asked to participate in a pilot study once the app has been developed. This iterative process allows for triangulation of the data.

### Safety Procedures

The end-user consultations were considered low risk, as participants were not asked to share their loss experiences directly but instead reflect on specifically helpful and unhelpful techniques and advice. Participants in both contexts were given informed consent sheets, which explained their rights to withdraw from the study at any time.

In the Swiss context, participants meet with the researcher before the focus group in one-on-one online sessions, where the content of the informed consent sheet is explained again; this includes an offer to speak to a clinical psychologist (CK) if needed. Participants are encouraged to ask questions. The interviews are carried out by a doctoral student with experience in mental health support.

In the Chinese context, participants also filled out an informed consent form. If distress is noticed during the interviews, participants will be guided through brief breathing exercises. Participants will additionally receive self-help resources as well as signposting after the interview.

### Data Analysis

All qualitative data will be transcribed in person (Chinese study) or by using an online transcription program (Swiss study). The data for both studies will be analyzed independently. The data will be analyzed using the framework method [69,70]. This method was chosen due to its highly structured and steps-based nature. This is particularly helpful for the iterative process, in which a clear overview of demands and ideas is required [69]. The steps of framework analysis apply to both contexts and will be used for both the expert interviews and the end-user interviews. Each set of interviews will be handled separately, as each group is asked different questions and builds on different preexisting data. We will first transcribe the data and then familiarize ourselves with the data by reading the transcripts or relistening to the audio files [69]. For the coding process, a combined approach mixing deductive and inductive approaches will be used. This approach was chosen because the study includes several prespecified questions and theoretical frameworks that will guide coding, such as CBT techniques, psychoeducational content, themes aligned with the dual process model of coping with bereavement, questions related to community connection, and culturally specific considerations (eg, the use of formal vs informal language in German, such as “Du” vs “Sie”). However, we do expect unpredicted codes to emerge, especially when speaking to end users or experts from different backgrounds. A second coder will independently code some of the interviews. Once this has been done, an analytical framework will be developed with specific codes and applied to all transcripts from the same context and category. We will use MAXQDA to code and chart the data. Data will be interpreted to generate insights for app development.

Both the end-user and expert insights will be used for the app. If conflicting information emerges between experts or end users, information will be discussed to develop a consensus within the research team. All analyses will be reported in line with the consolidated criteria for reporting qualitative research [71].

All participants (experts or end users) will be asked for their feedback on the app intervention.

## Results

### Overview

**Figure 2.**
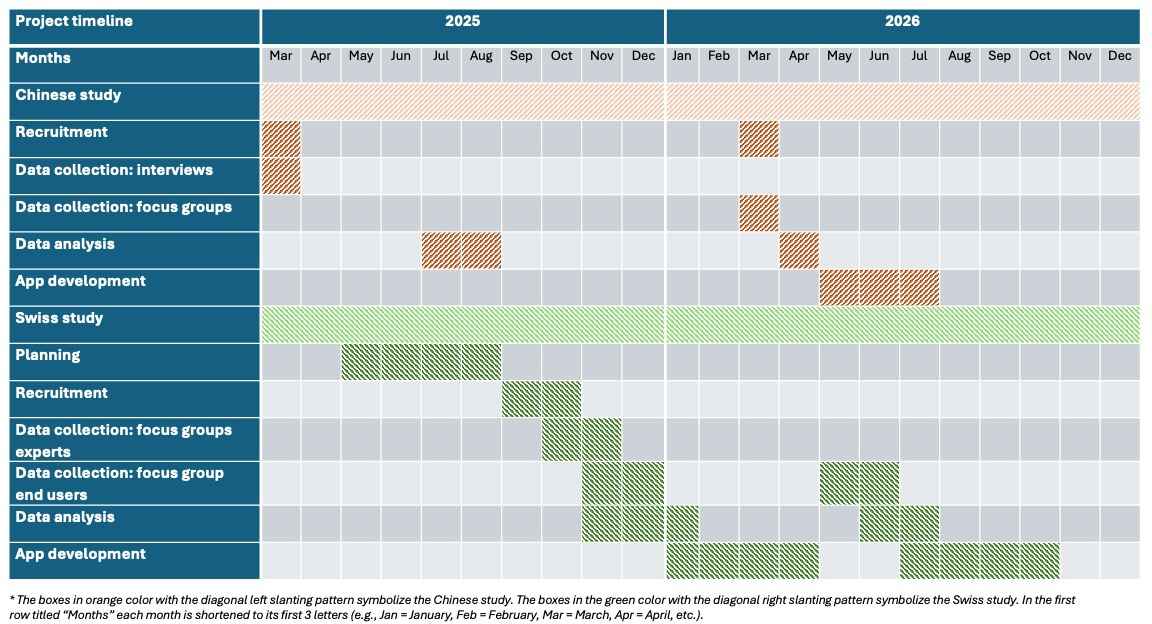
Project timeline.

As of September 2025, the first round of interviews with end users for the Chinese study has been completed, and all interviews have been transcribed. The Swiss study has begun recruitment for the expert interviews. Figure 2 depicts the project timeline.

### Current Status

#### Scoping

The scoping stage has already been conducted in both studies. In the Swiss context, this includes a systematic literature review of just-in-time adaptive interventions for common mental health disorders (currently in preparation; PROSPERO registration CRD42024611067).

For the Chinese context, a systematic review and meta-analysis of grief interventions in non-Western countries was conducted (currently in preparation; PROSPERO registration: CRD42024615519).

#### End-User Consultation

In the Chinese study, 17 one-on-one online interviews with bereaved adults have already been conducted, transcribed, and evaluated. We analyzed the data using a framework analysis approach. The analysis indicated that bereaved individuals commonly reported difficulties accepting the reality of the loss, fears of forgetting the deceased, sleep disturbances, and social withdrawal. Many described maintaining an emotional connection through interactions with the deceased’s belongings. Coping strategies were mainly social and behavioral in nature. Although most participants had not previously used digital support services, they expressed a strong preference for interventions that are flexible, easy to use, and personalized, with involvement from professionals and the incorporation of innovative features such as artificial intelligence. The interview findings have been prepared as a separate manuscript and are currently under review. The full findings will be published elsewhere at a later date.

#### EMA Items

While the Chinese EMA items are being translated and developed with the Chinese experts, the EMA items from the Swiss study are taken from an existing EMA study, which used German items [56].

### Expected Outcomes

The Chinese app is expected to be completed in 2026 and will primarily feature a collection of self-developed modules to support bereaved individuals. These modules are expected to include a community communication section, a memory recording section (which supports users to upload text, photos, and messages), and a daily data recording section (which records emotions, activities, etc).

We anticipate completing development of the Swiss app by autumn 2026. We expect to develop a mixture of background information, which is always available to the app users, and short microinterventions for daily use. m-Path additionally offers the option for users to save things such as pictures or messages to a library; this feature could be used as a memory wall. A library of all the microintervention content will also be included. The microinterventions (our EMI component) will be either text-based or audio-based.

## Discussion

This study presents the framework for the development of 2 EMIs for community grief support, one in China and one in Switzerland.

### Differences and Similarities in Approaches

#### Etic and Emic Approaches

Emic and etic approaches in cross-cultural research refer to whether behaviors are studied from within a culture, including culturally specific uniqueness (emic), or from outside a culture, identifying universal patterns (etic) [72,73]. In the context of app development, this distinction can be reflected in whether interventions are designed based on presumed universal mechanisms or are developed within a specific cultural context, taking into account unique needs, expressions, and demands. Nowadays, researchers recommend a mix of approaches to ensure that intervention content is evidence based as well as relevant and accurately adapted to the population [74]. The mobile health evidence reporting and assessment checklist [75] echoes this by including a recommendation to report user feedback, therefore emphasizing the importance of user involvement in academic research settings.

While both of our studies included etic and emic approaches, we encountered a difference in how much was known about both settings from an academic standpoint and how many “universally applicable” *etic* techniques have ever been evaluated in a Chinese (non-WEIRD) context. In comparison, in the Swiss study, the WEIRD-developed *etic* techniques could be presumed to have at least base-level cultural relevance, and the emic approach was more directed at understanding and incorporating the end-user perspective. Thus, the order of expert and end-user consultation was different in both studies. The Swiss study began by discussing existing interventions with experts and then presented the findings to the end users, whereas in the Chinese study, experience of grief was first established with the end users to allow for the cultural adaptation with the grief experts. This allowed for an understanding of both the cultural experience of grief and the perspective of end users.

#### Access to Support

The landscape of grief mental health support in China is sparse, with insufficient and slow service development [12,26]. Out of the few available clinicians, many are also untrained in grief therapy [26]. In addition, death literacy has been noted as lacking in China [76,77], which can hinder the use of both formal and informal bereavement care. Professional support for grief is sometimes perceived to be less effective than informal support by friends and family, and a strong underlying cultural belief remains that “time will heal” [26,78].

In China and many WEIRD countries, even when specialist support is available, it is difficult to access and is reserved for those with clinical levels of grief [23]. Many bereaved people report not receiving any or insufficient bereavement care [59,79,80]. In addition, bereaved people seem to access more readily available support such as peer support groups rather than psychotherapy [59,79]. Thus, online bereavement support may help reduce this gap by offering evidence-based, easily accessible, and structured support that can also address emotional barriers to help seeking, such as shame, vulnerability, and fear of being judged, which may otherwise discourage individuals from accessing face-to-face services [23,31].

### Grief-Specific EMI Challenges

Understanding the everyday experience and fluctuations of grief is still in its infancy. Grief symptoms have been found to fluctuate on a daily basis, with some symptoms showing bigger fluctuations (eg, sadness about the loss), and others presenting as more stable (eg, emotional numbness) [8]. In addition to their high fluctuations, grief symptoms are complex in nature. Symptoms such as yearning and longing are often “bittersweet,” encompassing both pain over the loss and positive memories [81,82]. This variation and complexity highlight why a variety of techniques, including loss and restoration-oriented work, are often used in grief interventions [9,51,60]. However, simmering this down into microinterventions and getting the correct balance of intervention strategies might present a challenge. Smyth and Ebner-Priemer [83] point out that much is unknown about which microinterventions are best suited to effectively target processes in a “just-in-time” manner. It will be important to understand what intensity of intervention people would find beneficial and acceptable in an app-based format and which components may be more appropriately delivered within clinical therapy.

### Grief as a Social Experience

Grief is an inherently cultural and social experience [84]. Sharing experiences helps bereavement to “flow” [85], and loss often presents a social void that needs to be filled [86]. In addition, people who enjoyed their social life more than usual were found to grieve less [86]. In line with this, an argument has been made to strengthen the social aspects of grieving by taking a community approach (eg, compassionate communities or compassionate neighbors), which mainly integrates at the palliative care level [87,88]. Interventions such as Grief Coach (also known as help texts) have tried to achieve social integration by allowing people to sign up their friends and family to receive SMS text messages on how to support the grieving person [46]. However, research in widowed adults has found that emotional loneliness in particular (ie, feeling alone even when surrounded by others) is more strongly associated with PGD symptoms than social loneliness [89]. This highlights the importance of addressing this sense of loneliness beyond merely increasing social contact.

For our intervention, we will include social support and community building as one of the topics to discuss with both experts and end users, to ascertain the best methods and levels of encouragement toward social activity and communication. This might be quite challenging depending on people’s social situations (eg, social isolation), as well as stigma and disenfranchisement associated with grief. The benefit of including an emic development approach will be the integration of what social support or communal activities are important to end users within the local cultural context.

### Next Steps

Once a beta version of the app is developed, we aim to conduct 2 pilot feasibility trials. The trials will use both EMA data and data from baseline and follow-up questionnaires, with the main aim of evaluating adherence and gathering feedback on app acceptability. EMI adherence rates vary greatly between different studies [90,91]. Adherence to the prompts (how many prompts are filled out and how many recommended tasks are conducted) is paramount in providing the estimated effects for mental health improvement. In addition, adherence rates can be used as an estimate for user experience and as feedback to further improve the apps. Both pilots will include participants suitable for interventions at tiers 1 and 2 of the bereavement care pyramid (no PGD diagnosis). In a next step, we aim to conduct larger-scale randomized controlled trials to investigate the efficacy of the apps and to ensure they are appropriately tested and evidence based. All study procedures will be preregistered on the Open Science Framework once ethical review has been completed.

### Strengths and Limitations

The strength of our protocol lies in the integration of diverse expert input and existing knowledge with end users’ lived experiences of bereavement and cultural context. This should help increase app acceptability and feasibility.

Another strength of these apps is the use of EMA data alongside our EMI study. These EMA data can enhance insight into participants’ experiences at the time of intervention delivery and help examine how these experiences relate to engagement and adherence (eg, compliance). EMA studies have been shown to be feasible and acceptable in bereaved populations [56,92].

One limitation of this protocol is inherent to the app development with m-Path, which somewhat limits the options for aesthetic app design. App aesthetics have previously been linked to consumer ratings in commercial mental health apps [93]. However, this aspect has not been prioritized in this protocol, where the main priority is translating grief support into an appropriate EMI format.

The other limitation is that, at this point in time, the outcome of the focus groups is unclear, and thus, the protocol might be adapted. Should this be the case, all changes will be specified in following publications.

In addition, no plan for the longevity of the interventions has been made. Thus, questions such as long-term cost assessments, app dissemination, data security agreements beyond research, and long-term maintenance are not yet addressed.

### Conclusions

This study presents a protocol for the development of 2 EMIs for grief. We discuss some similarities and differences as well as potential challenges for the development of the interventions.

## Supplementary material

10.2196/87021 Multimedia Appendix 1 m-Path security statement.

10.2196/87021 Multimedia Appendix 2 Interview guides.

## Abbreviations

CBT: cognitive behavioral therapy
EMA: ecological momentary assessment
EMI: ecological momentary intervention
PGD: prolonged grief disorder
WEIRD: Western, educated, industrialized, rich, and democratic

## References

[1] Knipscheer J, Sleijpen M, Frank L (2020). Prevalence of potentially traumatic events, other life events and subsequent reactions indicative for posttraumatic stress disorder in the Netherlands: a general population study based on the Trauma Screening Questionnaire. Int J Environ Res Public Health.

[2] Pop-Jordanova N (2021). Grief: aetiology, symptoms and management. Pril (Makedon Akad Nauk Umet Odd Med Nauki).

[3] Ergun TD, Ten Klooster PM, Bohlmeijer ET, Westerhof GJ, Franzen M, Lenferink LI (2025). Assessing prolonged grief symptoms using experience sampling methodology: the development of the prolonged grief symptoms - short ecological assessment (PGS-SEA) scale. Compr Psychiatry.

[4] Li J, Li Y, Wang Y, Jishi W, Fang J (2023). What we know about grief intervention: a bibliometric analysis. Front Psychiatry.

[5] Simon NM, Shear MK, Reynolds CF (2020). Commentary on evidence in support of a grief-related condition as a DSM diagnosis. Depress Anxiety.

[6] Arizmendi BJ, O’Connor MF (2015). What is “normal” in grief?. Aust Crit Care.

[7] Layne CM, Saltzman WR, Pynoos RS (2002). Grief reactions: a clinician’s perspective. Marriage Fam.

[8] Lenferink LI, Terbrack E, van Eersel JH, Zuidersma M, Franzen M, Riese H (2024). Fluctuations of prolonged grief disorder reactions in the daily life of bereaved people: an experience sampling study. Curr Psychol.

[9] Stroebe M, Schut H (1999). The dual process model of coping with bereavement: rationale and description. Death Stud.

[10] ICD-11 for mortality and morbidity statistics. World Health Organization.

[11] First MB, Yousif LH, Clarke DE, Wang PS, Gogtay N, Appelbaum PS (2022). DSM-5-TR: overview of what’s new and what’s changed. World Psychiatry.

[12] Yuan MD, Liu JF, Zhong BL (2024). Prevalence of prolonged grief disorder and its symptoms among bereaved individuals in China: a systematic review and meta‐analysis. Gen Psychiatry.

[13] Pan H, Liu F (2021). The prevalence of complicated grief among Chinese people at high risk: a systematic review and meta-analysis. Death Stud.

[14] Hilberdink CE, Ghainder K, Dubanchet A (2023). Bereavement issues and prolonged grief disorder: a global perspective. Glob Ment Health (Camb).

[15] Gureje O, Lewis-Fernandez R, Hall BJ, Reed GM (2019). Systematic inclusion of culture-related information in ICD-11. World Psychiatry.

[16] Gureje O, Lewis-Fernandez R, Hall BJ, Reed GM (2020). Cultural considerations in the classification of mental disorders: why and how in ICD-11. BMC Med.

[17] Xiu D, Maercker A, Woynar S, Geirhofer B, Yang Y, Jia X (2016). Features of prolonged grief symptoms in Chinese and Swiss bereaved parents. J Nerv Ment Dis.

[18] Hsu BY, Lu J, Barclay K, McMahon D, Stearns PN (2024). The Routledge History of Happiness.

[19] Li J, Prigerson HG (2016). Assessment and associated features of prolonged grief disorder among Chinese bereaved individuals. Compr Psychiatry.

[20] Ho SM, Chow AY, Chan CL, Tsui YK (2002). The assessment of grief among Hong Kong Chinese: a preliminary report. Death Stud.

[21] Xu X, Wen J, Qian W, Zhou N, Jiang W (2024). Living with grief and thriving after loss: a qualitative study of Chinese parents whose only child has died. Eur J Psychotraumatol.

[22] Aoun SM, Breen LJ, O’Connor M, Rumbold B, Nordstrom C (2012). A public health approach to bereavement support services in palliative care. Aust N Z J Public Health.

[23] Lenferink LI, de Keijser J, Eisma MC, Smid GE, Boelen PA (2021). Treatment gap in bereavement care: (online) bereavement support needs and use after traumatic loss. Clin Psychol Psychother.

[24] Harrop E, Goss S, Farnell D (2021). Support needs and barriers to accessing support: baseline results of a mixed-methods national survey of people bereaved during the COVID-19 pandemic. Palliat Med.

[25] Breen LJ, Moullin JC (2022). The value of implementation science in bridging the evidence gap in bereavement care. Death Stud.

[26] Tang S, Peng W, Qian X, Chen Y (2024). Healing grief - an online self-help intervention programme for bereaved Chinese with prolonged grief: study protocol for a randomised controlled trial. Eur J Psychotraumatol.

[27] Selman LE, Sutton E, Medeiros Mirra R (2023). “Sadly I think we are sort of still quite white, middle-class really” - inequities in access to bereavement support: Findings from a mixed methods study. Palliat Med.

[28] Jin W, Peng W, Tang S (2025). Acceptability and feasibility of an internet-based intervention for bereaved Chinese with prolonged grief: a mixed methods study. Eur J Psychotraumatol.

[29] Dilmaç JA (2018). The new forms of mourning: loss and exhibition of the death on the internet. Omega (Westport).

[30] Lüddeckens D (2018). Alternative death rituals in Switzerland: building a community of shared emotions and practices. J Contemp Relig.

[31] Eklund R, Eisma MC, Boelen PA, Arnberg FK, Sveen J (2021). Mobile app for prolonged grief among bereaved parents: study protocol for a randomised controlled trial. BMJ Open.

[32] Aeschlimann A, Reitsma L, Killikelly C, Smid GE, Comtesse H, Boelen PA (2025). Psychotherapy for Prolonged and Traumatic Grief.

[33] Zuelke AE, Luppa M, Löbner M (2021). Effectiveness and feasibility of internet-based interventions for grief after bereavement: systematic review and meta-analysis. JMIR Ment Health.

[34] Dominguez-Rodriguez A, Sanz-Gomez S, González Ramírez LP (2023). The efficacy and usability of an unguided web-based grief intervention for adults who lost a loved one during the COVID-19 pandemic: randomized controlled trial. J Med Internet Res.

[35] Finucane A, Canny A, Mair AP (2025). A rapid review of the evidence for online interventions for bereavement support. Palliat Med.

[36] Yao D, Qian F, Tung TH, Shi H, Bi D (2025). The effectiveness of web-based grief intervention for adults who lost a loved one: a systematic review and meta-analysis. BMC Palliat Care.

[37] Wagner B, Rosenberg N, Hofmann L, Maass U (2020). Web-based bereavement care: a systematic review and meta-analysis. Front Psychiatry.

[38] Heron KE, Smyth JM (2010). Ecological momentary interventions: incorporating mobile technology into psychosocial and health behaviour treatments. Br J Health Psychol.

[39] Meinlschmidt G, Lee JH, Stalujanis E (2016). Smartphone-based psychotherapeutic micro-interventions to improve mood in a real-world setting. Front Psychol.

[40] Balaskas A, Schueller SM, Cox AL, Doherty G (2021). Ecological momentary interventions for mental health: a scoping review. PLoS One.

[41] Lecomte T, Potvin S, Corbière M (2020). Mobile apps for mental health issues: meta-review of meta-analyses. JMIR Mhealth Uhealth.

[42] Versluis A, Verkuil B, Spinhoven P, van der Ploeg MM, Brosschot JF (2016). Changing mental health and positive psychological well-being using ecological momentary interventions: a systematic review and meta-analysis. J Med Internet Res.

[43] Marshall JM, Dunstan DA, Bartik W (2020). Apps with maps-anxiety and depression mobile apps with evidence-based frameworks: systematic search of major app stores. JMIR Ment Health.

[44] Levesque DA, Lunardini MM, Payne EL, Callison-Burch V (2025). Grief Coach, a text-based grief support intervention: acceptability among hospice family members. Omega (Westport).

[45] Help Texts.

[46] Levesque DA, Lunardini MM, Adams SN, Payne EL, Neumann BG (2025). *Grief Coach*: feasibility and acceptability of a text message program for bereavement support among grievers in the United Kingdom. Death Stud.

[47] Sveen J, Eisma MC, Boelen PA, Arnberg FK, Eklund R (2025). My Grief app for prolonged grief in bereaved parents: a randomised waitlist-controlled trial. Cogn Behav Ther.

[48] Eklund R, Eisma MC, Boelen PA, Arnberg FK, Sveen J (2022). My Grief app for prolonged grief in bereaved parents: a pilot study. Front Psychiatry.

[49] Mehrotra S, Tripathi R, Sengupta P (2025). Evaluating characteristics and quality of mental health apps available in app stores for Indian users: systematic app search and review. JMIR Mhealth Uhealth.

[50] Torous J, Linardon J, Goldberg SB (2025). The evolving field of digital mental health: current evidence and implementation issues for smartphone apps, generative artificial intelligence, and virtual reality. World Psychiatry.

[51] Boelen PA, de Keijser J, van den Hout MA, van den Bout J (2007). Treatment of complicated grief: a comparison between cognitive-behavioral therapy and supportive counseling. J Consult Clin Psychol.

[52] Komischke-Konnerup KB, Zachariae R, Boelen PA, Marello MM, O’Connor M (2024). Grief-focused cognitive behavioral therapies for prolonged grief symptoms: a systematic review and meta-analysis. J Consult Clin Psychol.

[53] Haber Y, Hadar Shoval D, Levkovich I (2025). The externalization of internal experiences in psychotherapy through generative artificial intelligence: a theoretical, clinical, and ethical analysis. Front Digit Health.

[54] Eisma MC, van der Laan J, Franzen M, aan het Rot M (2024). Does self-monitoring reduce prolonged grief symptoms? Pre-post analyses of a diary study. J Loss Trauma.

[55] Bakker D, Rickard N (2018). Engagement in mobile phone app for self-monitoring of emotional wellbeing predicts changes in mental health: MoodPrism. J Affect Disord.

[56] Lenferink LI, van Eersel JH, Franzen M (2022). Is it acceptable and feasible to measure prolonged grief disorder symptoms in daily life using experience sampling methodology?. Compr Psychiatry.

[57] Boelen PA, van den Hout MA, van den Bout J (2006). A cognitive-behavioral conceptualization of complicated grief. Clin Psychol.

[58] Smid GE, Groen S, Comtesse H, Smid GE, Comtesse H, Boelen PA (2025). Psychotherapy for Prolonged and Traumatic Grief: A Guide for Mental Health Professionals.

[59] Aoun SM, Breen LJ, Howting DA, Rumbold B, McNamara B, Hegney D (2015). Who needs bereavement support? A population based survey of bereavement risk and support need. PLoS One.

[60] Komischke-Konnerup KB, O’Connor M, Hoijtink H, Boelen PA (2025). Cognitive-behavioral therapy for complicated grief reactions: treatment protocol and preliminary findings from a naturalistic setting. Cognit Behav Pract.

[61] m-Path.

[62] Mestdagh M, Verdonck S, Piot M (2023). m-Path: an easy-to-use and highly tailorable platform for ecological momentary assessment and intervention in behavioral research and clinical practice. Front Digit Health.

[63] Saparamadu AA, Fernando P, Zeng P (2021). User-centered design process of an mHealth app for health professionals: case study. JMIR Mhealth Uhealth.

[64] Birrell L, Furneaux-Bate A, Debenham J, Spallek S, Newton N, Chapman C (2022). Development of a peer support mobile app and web-based lesson for adolescent mental health (Mind Your Mate): user-centered design approach. JMIR Form Res.

[65] Vial S, Boudhraâ S, Dumont M (2022). Human-centered design approaches in digital mental health interventions: exploratory mapping review. JMIR Ment Health.

[66] Ahluwalia S, Bandini J, Maglione M (2026). Care of bereaved persons: a systematic review. Ann Intern Med.

[67] Killikelly C, Christen LM, Groen S (2025). Feasibility, acceptability and clinical utility of the bereavement and grief cultural formulation interview for prolonged grief disorder. Cult Med Psychiatry.

[68] Killikelly C, Zhou N, Merzhvynska M (2020). Development of the International Prolonged Grief Disorder Scale for the ICD-11: measurement of core symptoms and culture items adapted for Chinese and German-speaking samples. J Affect Disord.

[69] Gale NK, Heath G, Cameron E, Rashid S, Redwood S (2013). Using the framework method for the analysis of qualitative data in multi-disciplinary health research. BMC Med Res Methodol.

[70] Ritchie J, Ormston R, McNaughton Nicholls C, Lewis J (2013). Qualitative Research Practice: A Guide for Social Science Students and Researchers.

[71] Tong A, Sainsbury P, Craig J (2007). Consolidated criteria for reporting qualitative research (COREQ): a 32-item checklist for interviews and focus groups. Int J Qual Health Care.

[72] Fernando GA (2012). The roads less traveled: mapping some pathways on the global mental health research roadmap. Transcult Psychiatry.

[73] Killikelly C, Bauer S, Maercker A (2018). The assessment of grief in refugees and post-conflict survivors: a narrative review of etic and emic research. Front Psychol.

[74] Wu X, Xu L, Li P, Tang T, Huang C (2022). Multipurpose mobile apps for mental health in Chinese app stores: content analysis and quality evaluation. JMIR Mhealth Uhealth.

[75] Agarwal S, LeFevre AE, Lee J (2016). Guidelines for reporting of health interventions using mobile phones: mobile health (mHealth) evidence reporting and assessment (mERA) checklist. BMJ.

[76] Li X, Che SL, Zhu M, Ng WI (2024). What we learnt from parents’ death experience: a cross-sectional study of death literacy and parent’s death quality among adult children in China. Pall Supp Care.

[77] Shu W, Miao Q, Feng J, Liang G, Zhang J, Zhang J (2023). Exploring the needs and barriers for death education in China: getting answers from heart transplant recipients’ inner experience of death. Front Public Health.

[78] Tang S, Chow AY, Breen LJ, Prigerson HG (2020). Can grief be a mental disorder? An online survey on public opinion in mainland China. Death Stud.

[79] Lichtenthal WG, Corner GW, Sweeney CR (2015). Mental health services for parents who lost a child to cancer: if we build them, will they come?. J Clin Oncol.

[80] Maccallum F, Breen LJ, Ivynian S, DiGiacomo M, Luckett T, Lobb EA (2025). Prolonged grief reactions and help-seeking in bereaved adults during the COVID-19 pandemic. J Affect Disord.

[81] Riches G, Pearce C, Komaromy C (2021). Narratives of Parental Death, Dying and Bereavement.

[82] Baddeley JL, Williams JL, Rynearson T, Correa F, Saindon C, Rheingold AA (2015). Death thoughts and images in treatment-seekers after violent loss. Death Stud.

[83] Smyth JM, Ebner-Priemer U (2025). Dispelling “pleasing myths” about the integration of ecological momentary assessment and intervention into clinical research and practice. World Psychiatry.

[84] Brinkmann S, Kofod EH (2018). Grief as an extended emotion. Cult Psychol.

[85] Henoch I, Berg C, Benkel I (2016). The shared experience help the bereavement to flow: a family support group evaluation. Am J Hosp Palliat Care.

[86] Specker P, Pociūnaitė-Ott J, Rosenblum AL (2025). The association between one’s social life and symptoms of prolonged grief following a traumatic loss: an ecological momentary assessment study. Eur J Psychotraumatol.

[87] Noonan K, Rumbold B, Aoun SM (2023). Compassionate community connectors: a distinct form of end-of-life volunteering. Prog Palliat Care.

[88] Sallnow L, Bunnin A, Richardson H, Wegleitner K, Heimerl K, Kellehear A (2015). Compassionate Communities.

[89] Vedder A, O’Connor M, Boelen PA (2025). Emotional vs. social loneliness and prolonged grief: a random-intercept cross-lagged panel model. Eur J Psychotraumatol.

[90] Marciniak MA, Shanahan L, Rohde J (2020). Standalone smartphone cognitive behavioral therapy-based ecological momentary interventions to increase mental health: narrative review. JMIR Mhealth Uhealth.

[91] Dao KP, De Cocker K, Tong HL, Kocaballi AB, Chow C, Laranjo L (2021). Smartphone-delivered ecological momentary interventions based on ecological momentary assessments to promote health behaviors: systematic review and adapted checklist for reporting ecological momentary assessment and intervention studies. JMIR Mhealth Uhealth.

[92] Mintz EH, Toner ER, Skolnik AM (2026). Ecological momentary assessment in prolonged grief research: feasibility, acceptability, and measurement reactivity. Death Stud.

[93] Lau N, O’Daffer A, Yi-Frazier JP, Rosenberg AR (2021). Popular evidence-based commercial mental health apps: analysis of engagement, functionality, aesthetics, and information quality. JMIR Mhealth Uhealth.

