# Supplementary material for: Mobile-Based Ecological Momentary Interventions for Grief in China and Switzerland: Protocol for a Collaborative and Iterative Qualitative App Development Study
Source: JMIR Res Protoc. 2026 Jun 9;15:e87021. doi: 10.2196/87021 (PMC13249120; doi:10.2196/87021)
Supplement: Multimedia Appendix 1 [file resprot-v15-e87021-s001.pdf]

Security Statement

This Security Statement applies to the products, services, websites and apps offered by m-Path. In this statement we will refer to the smartphone app that is used by clients and participants as the m-Path app (See here for Android and here for iOS). We will refer to the website (see here), used by practitioners and or researchers to create questionnaires and to analyze the data as the m-Path online dashboard.

Both the m-Path app as well as the m-Path online dashboard processes potentially sensitive information. We value the trust our users, both clients as well as practitioners, place in us by using our app and online dashboard and are committed to protect and secure the data of our them. The privacy policy of both the m-Path app as well as the m-Path online dashboard provides further details on how we handle your data.

m-Path Servers

m-Path is hosted on Microsoft Azure servers in Frankfurt, Germany. Microsoft Azure servers are compliant to many international standards (e.g. ISO ISO 27001 and SOC 2): https://learn.microsoft.com/en-us/azure/compliance/

Physical Security

Microsoft designs, builds, and operates datacenters in a way that strictly controls physical access to the areas where data is stored. Microsoft understands the importance of protecting data, and is committed to helping secure the datacenters that contain data. They have an entire division devoted to designing, building, and operating the physical facilities supporting Azure. This team is invested in maintaining state-of-the-art physical security. More information can be found here: https://learn.microsoft.com/en-us/azure/security/fundamentals/physical-security

Firewall

Our servers use Azure Front Door to Safeguard our platform and content. Azure Front Door dan be used for web application firewall (WAF), DDoS protection, and bot protection (see https://azure.microsoft.com/en-us/products/frontdoor)

Communication security

All communications between the m-Path app and the Microsoft Azure servers and between the m-Path online dashboard and the Microsoft Azure servers are done using a secure HTTPS connection. All the questions asked by the practitioner (through the m-Path online dashboard) and answers given by the client (through the m-Path app) are sent to the server using this secure HTTPS connection. HTTPS stands for Hypertext Transfer Protocol Secure. This protocol was designed to guarantee secure communications over the internet. Specifically, HTTPS is used for authentication and privacy. First, HTTPS is used to authenticate the server (using a list of well-known certificate authorities), to make sure data is not send to a fake server. Additionally, HTTPS is used to authenticate the data itself, to confirm that they are not modified during transmission. Second, HTTPS is used to ensure privacy of the communication: the communication between the m-Path app and server, and the communication between the online dashboard and server is fully encrypted (TLS 1.2, AES with 256 bit encryption; ECDH with 256 bit exchange) so that an intercepted transmission cannot be deciphered by another party.

Encryption at rest

The data on our servers is encrypted at rest with AES 256-bit encryption via Azure Storage encryption (service managed keys).

Local storage in m-Path app

All data on the m-Path app (e.g., questions and answers) are stored in a protected folder on the smartphone of the user which can only be accessed by the m-Path app. This folder cannot be accessed by other apps.

To enhance data security and to prevent data leakage we apply an application-layer encryption. All answers given to questionnaires, all downloaded questionnaires, added and removed practitioners, personal information (i.e. alias), text information of pluspoints, options and notes are stored on the phone using AES 256 bit-encryption with PKCS7 padding. This means that the stored data itself consists of bytes without any meaning.

Security m-Path app code

Flutter, the development kit which is used to develop the m-Path app created builds on the Android and iOS app sandbox environments, so the m-Path app has the inherent security of native Android and iOS apps. Sandbox environments isolate apps from each other and protect apps and the system from malicious apps. All m-Path app code is written in Dart, a type-safe language to prevent input validation issues, one of the most common security problems. Additionally, we don't load dynamical code in the m-Path app to prevent code injection or code tampering.

We also use code obfuscation for the m-Path app to create machine code that is almost impossible to understand for hackers. Code obfuscation conceals the logic and purpose of the programming code. This makes the m-Path app much more difficult to reverse engineer, which protects us from the discovery of unforeseen vulnerabilities in the tool. Specifically, we obfuscate all the Dart code (Dart is the programming language used to program m-Path) and Java code (additional code used in the Android environment).

Pseudonymization participant data

Participants (m-Path app users) don't need to give their name, email, phone number nor internet account (e.g. Facebook or Google) to use the m-Path app. Researchers and practitioners can identify participants using invitation codes and a personal reference table that links invitation codes to real participants. This reference table is not accessible to m-Path, making it impossible to identify participants using only the data available on the platform. This means that that participant data is pseudonymized. If researchers (m-Path dashboard users) choose to ask about data that identifies the participant (e.g. name or address or e-mail) in a questionnaire, the data is no longer pseudonymized.

Development

Our development team employs secure coding techniques and best practices, focused around the OWASP Top Ten and the OWASP Mobile Application Security Verification Standard. Development, testing, and production environments are separated. All changes are reviewed by the whole team and logged for performance, audit, and forensic purposes prior to deployment into the production environment. To ensure users are not using deprecated versions of our software, we have a mechanism to enforce updates for both the m-Path app as well as the m-Path online dashboard.

Patch management

m-Path servers use patch management that is done automatically by Azure, for the Azure App Service (website, dashboard, manual, API) as well as the Azure Database for MySQL.

Breach Notification

Notwithstanding our best efforts, it is impossible to guarantee absolute security in data transmission or storage. If we learn of a security breach, we will alert affected users so they can take appropriate protective measures.

Business Continuity

We use zone-redundant HA architecture (https://learn.microsoft.com/en-us/azure/mysql/flexible-server/concepts-high-availability#zone-redundant-ha-architecture) which ensures a standby replica server in another availability zone. If there is a failover, the standby replica is activated. Additionally, our servers are back upped daily and we regularly test our procedure for service restoration.

Information Security Aspects of Business Continuity Management

Our databases are backed up on a rotating hourly, daily and weekly basis (maximum retainment of one month).

m-Path dashboard account security

m-Path dashboard users are obligated to use a strong password (at least 8 characters including a number and a symbol) when creating an account. Additionally, they can enable two-factor authentication.

Your Responsibilities

Keeping m-Path secure also requires the users to take appropriate measures. m-Path app users should not use jailbroken devices as this can impair the security of the app. m-Path online dashboard users should use secure passwords and keep them safe. Both m-Path app users as well as m-Path online dashboard users should ensure that they have sufficient security on their own systems.

Logging and Monitoring

Activity on our servers and m-Path services in particular is logged for purposes of troubleshooting, security reviews and load analysis. We have the following logs:

- **Azure Audit Logs:** These provide a detailed history of management and operational activities within the Azure server environment, such as server creation, configuration changes, resource scaling, and user access events. These logs are captured primarily in Azure Activity Logs.
- **MySQL Audit Logs:** These capture database-specific activities, including connection attempts, permission and role changes, schema modifications. Query executions can be logged temporarily.
- **Application Logs:** Logs generated by applications running on the server (e.g., HTTP request logs in Azure App Service) are kept for end-to-end request tracing and diagnosing application behavior.
- **Code Changes:** Changes to application or infrastructure code are typically tracked through version control systems like GitHub or Azure Repos.
- **m-Path platform logs**: we also log activities from researchers or practitioners such as when a researcher logs into m-Path.

Access Control

Access control is a fundamental technical and organizational measure for any SaaS company, ensuring that only authorized individuals can access specific systems, data, or functionalities based on their roles and responsibilities. Access to m-Path's technology resources is only permitted through secure connections (e.g., VPN, SSH). We are implementing role-based access control (RBAC) which allows us to assign permissions based on job functions, minimizing the risk of unauthorized access. The principle of least privilege further strengthens our security by limiting user access to only what is necessary for their tasks. Multi-factor authentication (MFA) is also used, adding an additional layer of verification to reduce the likelihood of credential compromise.

Penetration tests

As part of our ongoing commitment to security, we conduct comprehensive penetration testing on an annual basis. We take the results of these assessments seriously and use them to continuously improve our security posture, aligning with industry best practices and regulatory requirements.

Employee management

We have onboarding and offboarding procedures to securely manage user lifecycle and asset control. All employees managing the backend (servers and database) are regularly educated regarding phishing, data handling, etc..
