# Supplementary material for: Mobile-Based Ecological Momentary Interventions for Grief in China and Switzerland: Protocol for a Collaborative and Iterative Qualitative App Development Study
Source: JMIR Res Protoc. 2026 Jun 9;15:e87021. doi: 10.2196/87021 (PMC13249120; doi:10.2196/87021)
Supplement: Multimedia Appendix 2 [file resprot-v15-e87021-s002.docx]

Expert interview China

1. **Interview questions:**

**App related questions**

*(after introduce the app)Evaluation of the initial version of the application*

-Do you think the modules we've designed so far are effective?

-How should we improve each module?

-What modules do you think we need to add?

-What do you think we should pay attention to when implementing this application?

**Grief related questions**

*Opinions for bereavement digital support*

*Who would benefit best from digital format of support? Who would not benefit?*

*What type of support might be best offered in a digital format, or in a face to face format? What type of exercises? Interventions, skills? Modules would be particularly aligned with digital format*

-If there is a grief digital support, what do you think is needed? Why is a digital format of grief support needed? What does a digital format allow(afford) patients? What could be some advantages of digital formats for therapy or assessment compared to face to face?

Are there any culture-specific factors or considerations that are particularly important when working with bereaved individuals in the Chinese context?

**EMA related questions**

-We plan to monitor participants' emotional components such as yearning, sadness, anger, and numbness daily. Which item are we missing? And which symptoms do you think will fluctuate within a day/hourly according to your knowledge?

-After collecting EMA information, do you think we should provide treatment modules randomly or provide them selectively based on the EMA information?

-How do you think we should schedule our daily EMA tasks?

-What are some basic questions that we absolutely need to set?

-What should we pay attention to when setting questions? For example, the number and length of questions?

-What aspects should be considered when collecting data to benefit subsequent data analysis?

Expert Interview Switzerland

- Please describe your expertise with grief
- What are is the most important information that should be included in a self-help app for grief support? What are the most important messages?
- What are the most important exercises / skills to include in the app?
  - How would you suggest breaking these into smaller exercises to fit the EMI format?
- We know that grief can be a very lonely time, what can we include in the app to encourage people to connect to others or to their community?
- What uniquely Swiss factors should we consider? What uniquely Swiss aspects do you encounter in your work?
- What services should people be aware of?
- What other signposting or resources should the app include?
- What would make you recommend a self-help app for grief to someone?
- Is there anything else you think is important for us to know?
